# SARS-CoV-2 in Asthmatic Children: Same Consequences in Different Endotypes?

**DOI:** 10.3390/metabo15060406

**Published:** 2025-06-16

**Authors:** Alice Bosco, Vassilios Fanos, Serena Bosone, Valeria Incandela, Federica La Ciacera, Angelica Dessì

**Affiliations:** Neonatal Intensive Care Unit, AOU Cagliari, Department of Surgical Sciences, University of Cagliari, 09124 Cagliari, Italy; vafanos@tiscali.it (V.F.); s.bosone@studenti.unica.it (S.B.); v.incandela@studenti.unica.it (V.I.); f.laciacera@studenti.unica.it (F.L.C.); angelicadessi@unica.it (A.D.)

**Keywords:** pediatric asthma, SARS-CoV-2, asthma endotypes, metabolomics, obesity, risk stratification, precision medicine

## Abstract

During the early stages of the severe acute respiratory syndrome coronavirus 2 (SARS-CoV-2) pandemic, concerns arose regarding the susceptibility of asthmatic children, one of the most common chronic conditions in childhood and a major cause of hospitalization in pediatric settings. Unexpectedly, evidences showed milder clinical courses and fewer asthma exacerbations in these patients, even if cases of critical and fatal infection, often related to specific clinical features of the patient, are not negligible. In this regard, obesity is considered not only an important comorbidity in patients with difficult-to-treat asthma but also a risk factor for more severe forms of COVID-19. These observations are of even greater concern in the context of an increase in childhood obesity that began even before the SARS-CoV-2 pandemic and has continued also as a consequence of it. Given asthma’s heterogeneity, especially in children, an endotype-based approach is crucial. This is possible through a detailed analysis of the complex metabolic pathways that correlate asthma, COVID-19 infection and obesity thanks to new high-through-put technologies, especially metabolomics, which with minimally invasive sampling, including on exhaled breath condensate (EBC), can provide precise and unbiased evidence in support of existing endotypes, making it possible to identify not only the most vulnerable individuals and thus risk stratification through specific biomarkers, but also new molecular and therapeutic targets. This review explores asthma endotypes by highlighting their shared immunometabolic pathways with COVID-19. Findings suggest that metabolomics could enable more accurate risk stratification and guide personalized interventions during viral pandemics, especially in the presence of relevant comorbidities such as obesity.

## 1. Introduction

As a result of the spread of severe acute respiratory syndrome coronavirus 2 (SARS-CoV-2) in December 2019, the first pediatric cases were reported as early as January 2020 [1,2]. During the first pandemic phase, there was great concern about the risk of infection in children with asthma, especially due to the classification of asthma disease by the Centers for Disease Control and Prevention (CDC) as a risk factor for severe forms of COVID-19 [3]. Moreover, asthma is one of the most common chronic conditions in childhood and is a major cause of hospitalization in pediatric patients. Given COVID-19’s respiratory tropism, chronic lung diseases, especially asthma, were reasonably considered risk factors for severe infection in children [4].

To date, the evidence supporting the notion that asthma is not a single entity but rather a very complex biological network of distinct and interconnected inflammatory pathways is now robust and numerous. Indeed, asthma is characterized by different mechanistic pathways (endotypes) and variable clinical presentations (phenotypes), the precise identification of which is fundamental not only for its correct management but also for its therapeutic and prognostic implications [5].

Similarly to what was observed in children and adolescents where, on the whole, less severe clinical pictures emerged and the course of the disease was generally favorable compared to adults [6,7], in pediatric asthma patients the initial data were also positive. In fact, during the early stages of the pandemic, there was a noticeable reduction in emergency room visits. Asthma exacerbations and bronchospasm-related hospitalizations also declined [8,9]. These observations are also reflected in the data relating to the 2003 SARS-CoV epidemic, during which asthma was not identified as a risk factor in children [10]. Nevertheless, this phenomenon would be mainly attributable to the lower exposure to environmental and infectious triggers due to the containment measures, rather than to an intrinsic protective effect of asthma. Moreover, with the emergence of new viral variants, the possibility has emerged that the susceptibility of asthmatic children to SARS-CoV-2 infection varies according to the virological characteristics of the children themselves [11,12].

Furthermore, scientific evidence has now shown an increase in the severity and mortality of COVID-19 in the presence of pre-existing comorbidities, including obesity, making it necessary to investigate the ways in which these conditions and COVID-19 interact at a molecular level in order to develop increasingly personalized management strategies. In fact, despite the encouraging data regarding SARS-CoV-2 infection in asthmatic children, cases of increased risk of serious illness or death have been observed, especially in correlation with specific clinical characteristics [8,9]. In this regard, overweight and obesity are considered important comorbidities even in patients with difficult-to-treat asthma, supporting a specific phenotype of the disease, which differs from the phenotype considered most common in the pediatric population [13]. These observations take on even greater importance in the context of increasing childhood obesity that began even before the SARS-CoV-2 pandemic. However, the consequences of the pandemic, resulting in small increases in weight, BMI and the prevalence of obesity in children, in each case potentially clinically significant, must be added [14,15].

The importance of investigating the impact of COVID-19 on the different pediatric asthma endotypes therefore clearly emerges. This approach may in fact allow the identification of potential shared pathophysiological mechanisms also capable of implementing risk stratification and guiding personalized preventive and therapeutic interventions. In this regard, modern high-throughput technologies, in particular metabolomics, represent extremely promising tools to characterize the endotypes underlying the different clinical phenotypes of asthma [16], especially through the use of non-invasive matrices such as exhaled breath condensate (EBC) [17].

The aim of this narrative review is to provide an integrative overview of pediatric asthma endotypes in the context of SARS-CoV-2 infection, with a focus on shared immunological and metabolic mechanisms that may influence clinical vulnerability. Particular attention is given to the potential role of metabolomics in supporting endotype-based risk stratification and guiding personalized interventions. All peer-reviewed articles concerning the endotyping of pediatric asthma through metabolomic analysis were included.

## 2. Pediatric Asthma Endotypes

Asthma is characterized by a common symptomatology but very different responses to the same therapeutic interventions. This requires a broader assessment that considers its complexity in terms of severity, natural history and therapeutic efficacy, also useful for risk stratification in the presence of infectious diseases (such as COVID-19) and/or comorbidities. It is therefore necessary to focus on the presence of different endotypes, able to describe the distinct pathophysiological mechanisms at the cellular and molecular level of each asthmatic phenotype. In fact, the phenotype only describes the observable characteristics of the disease, such as age of onset, symptoms, response to treatment and any comorbidities. On the other hand, the endotype represents a subcategory of the disease defined by distinct molecular, immunological and physiopathological mechanisms [18,19]. It is therefore necessary to go beyond the phenotyping of allergic or non-allergic asthma in favor of a more complete approach that allows us to define both the clinical characteristics and the underlying endotype [20].

Hence, in order to improve the endotyping of young asthma patients, which to date is often mainly based on clinical features and lab parameters, it could be useful to deepen molecular analysis and extend new high-throughput technologies. In fact, omic approaches, including metabolomics and microbiomics, with minimally invasive sampling, have provided accurate and impartial evidence to support existing endotypes but have also suggested the conceptualization of new endotypes [16,21,22,23], together with the identification of biomarkers capable of predicting response to treatment and disease progression [21,22,23,24]. Indeed, in recent years, thanks to a high-throughput approach, it has been possible to identify numerous pediatric asthmatic endotypes, beyond the traditional Th2-high and Th2-low dichotomy, which nevertheless remains a point of reference in the immunological classification of asthma.

It has therefore emerged that the same Th2-high eosinophilic phenotype differentiates into at least three distinct clusters. The first is represented by mild atopic asthma with early onset, a low rate of exacerbations and preserved lung function. The second is persistent atopic asthma of moderate severity, with a medium rate of exacerbations, reduced lung function and severe hyperreactivity. Finally, there is severe, highly atopic asthma, with significant symptoms and the need for intensive drug treatment, characterized by severe hyperreactivity and compromised lung function [5,20]. Another feature of Th2-high endotype is a reduced production of type I and III interferons (IFN-I, IFN-III) by airway epithelial and dendritic cells, which correlates with possible impairment of innate antiviral immunity [5].

Then there is the Th2-low endotype, which presents in a non-eosinophilic form, frequently neutrophilic or paucigranulocytic. The paucigranulocytic variant is characterized by a low degree of inflammation, remodeling of the smooth muscle of the airways, bronchial hyperreactivity and persistent airflow limitation. The neutrophilic form, on the other hand, can include both mild asthma with early onset and no atopic factors, with normal lung function, and severe persistent non-allergic forms, poorly controlled, with high hyperreactivity, a high rate of exacerbations and frequent association with obesity [20,25]. In general, the Th2-low endotype is also characterized by a deficient IFN-I and IFN-III response, with impaired efficacy of the innate antiviral response and increased susceptibility to viral infections. At the same time, an increase in IFN-γ (type II) and cytokines such as IL-12, IL-17 and TNF-α has been observed, reflecting the joint activation of the Th1 and Th17 axes. This immunological make-up contributes to the onset of chronic, neutrophilic inflammation that is poorly responsive to corticosteroid therapy [5,20].

Furthermore, with the constant increase in the prevalence of obesity in children, concerns have arisen regarding the increase in less common asthmatic endotypes in the pediatric population. In fact, specific pediatric obesity-related phenotypes with peculiar clinical characteristics have been described, supported by distinct immunological endotypes. One is characterized by high levels of Th2 and eosinophilic inflammation and a second by low Th2 inflammation but high neutrophilic inflammation, associated with a later onset [26,27]. This latter profile, characterized by a low-grade systemic inflammatory state typical of obesity, is supported by the activation of M1 macrophages, responsible for the production of IL-6, TNF-α and IL-1β and the triggering of Th1 and Th17 cells, as the basis of neutrophilic inflammation. Although a direct causality between the two conditions has not yet been demonstrated, this particular obesity-related endotype suggests that excess weight may precede the development of asthma [28]. However, obesity can also coexist with a Th2-type inflammatory response in atopic individuals, delineating two distinct obesity-related endotypes. In both cases, systemic inflammation can lead to increased deposition of extracellular matrix and proliferation of smooth muscle cells, contributing to bronchial remodeling and hyperreactivity [29].

Other endotypes are also recognized, including the viral-induced endotype, which is frequent in preschool children with episodic wheezing, related to respiratory infections. In this case, neutrophilic inflammation may emerge in the acute phase as a response to viral infections, in a context where the interferon response is often immature or absent in younger children, contributing to epithelial dysfunction and recurrence of wheezing [20].

Finally, in cases of severe treatment-resistant asthma (STRA), an eosinophilic endotype is recognized with marked bronchial remodeling and severe hyperreactivity, often associated with high concentrations of IL-33 and the presence of innate lymphoid cells 2 (ILC2). This profile is often accompanied by a persistent Th2 response, immune dysregulation and reduced sensitivity to steroids, which require management with targeted biological drugs and intensive monitoring [20].

The most salient clinical–immunological characteristics of the main pediatric asthmatic endotypes are summarized in Table 1.

These new categorizations of asthma can, however, be further refined by modern high-throughput technologies, capable of providing unbiased molecular evidence for a precise and accurate definition of existing endotypes but also to delineate new ones [16,29].

## 3. Metabolomics Endotyping of Asthmatic Children

A greater understanding of the different asthmatic endotypes can be acquired thanks to the use of high-throughput omic technologies [21,30,31,32,33,34,35,36,37,38,39,40,41,42,43,44,45,46]. In this regard, metabolomics, through the study of metabolites—small molecules present in a biological system—provides a snapshot of the genome, transcriptome and proteome, and their interactions with the environment, highlighting the molecular mechanisms involved in the event of dysregulation. This approach overcomes the limitations of current asthma guidelines, which are based on a clinical classification unable to capture the extreme heterogeneity of the disease, resulting in suboptimal treatment strategies for some individuals. Furthermore, through metabolomics it is possible not only to highlight the altered molecular pathways on which to focus therapeutic intervention, but also to identify unique markers of the disease [35,36].

The metabolomics studies that have focused on endotyping childhood asthma are shown in Table 2, Table 3 and Table 4 and present different approaches based on the type of biological sample analyzed.

An initial attempt at endotyping asthmatic children was made in 2012 by Carraro et al. [30]. They conducted a cross-sectional study on 42 asthmatic children (aged between 8 and 17 years), of which 31 had non-severe asthma (whether or not treated with inhaled steroids), 11 had severe asthma and 15 were controls. The EBC was studied through metabolomic analysis conducted with mass spectrometry (NMR). The results showed a clear distinction of different biochemical-metabolic profiles in asthmatic children, through metabolites related to retinoic acid, adenosine and vitamin D, together with a clear discrimination of the metabolic phenotype of severe asthma [30]. Another EBC analysis was conducted by Sinha et al. [31] using NMR combined with machine learning (ML). They studied 89 asthmatic subjects from a prospective cohort and 20 healthy controls. The results of the research highlighted the efficiency of the NMR spectra of the EBC combined with ML in distinguishing asthmatics from healthy controls and delineating three distinct clusters in the asthmatic group, with relevant clinical and chemical differences [31]. Despite the growing interest in the use of non-invasive matrices such as EBC in the pediatric population, the studies conducted so far are preliminary, limited both by numerically small samples and by the absence of comparisons between the different clusters revealed, thus making it impossible to identify replicable and clinically significant endotypes. Nevertheless, these results clearly demonstrated the presence of a unique inflammatory and oxidative state in the various pediatric asthmatic endotypes.

A larger number of studies have instead concentrated on the analysis of plasma [32,33,34,35,36,38] and serum [21,39,40,41,42]. Using NMR, Cottrill et al. [32] analyzed the plasma extracted from 4 cohorts of pediatric asthma (215 subjects in total, of which 41 with asthma with a tendency to exacerbate). The results showed the presence of 32 unique serum metabolites, independent of the cohort, which distinguished asthmatic children with exacerbations from those without exacerbations. The main metabolic alterations involved the metabolism of three amino acids: arginine, lysine and methionine [32]. Fitzpatrick et al. [33] focused on the metabolomic analysis of patients with severe asthma, conducting a comparative analysis of the plasma of children with severe asthma treated with high-dose inhaled corticosteroids (ICS) and long-acting beta (β) agonists compared to children with mild–moderate asthma treated with ICS or a combined ICS/long-acting β agonist therapy. This research revealed the presence of two distinctive metabolic pathways associated with oxidative stress that are characteristic of children with severe asthma. The first involves the glycine, serine, and threonine pathways, while the second involves the N-acylethanolamine and N-acyltransferase routes. The authors emphasized the possible role of oxidative stress in the low sensitivity to corticosteroids in severe asthma, highlighting possible new therapeutic targets [33]. Another plasma metabolomic analysis was conducted by Papamichael et al. [34] through targeted GC-MS of the fatty acids of 64 children with a “mild-asthma” phenotype, revealing the presence of 25 unique plasma fatty acids in asthmatic children. Specifically, thanks to linear regression, the association between these lipid biomarkers and lung function indexes was analyzed. This allowed them to show that linoleic, oleic, erucic, cis-11-eicosenoic and arachidic acids were significantly associated with poorer asthma control and lower lung function in the group of overweight or obese children, while other specific associations emerged in the normal weight group (stearic and arachidic acids). The authors therefore concluded that through a nutritional intervention targeted according to the specific endotype it would be possible to optimize asthma control, lung function and therapeutic response in children [34]. Kelly et al. [35], in 2017, performed a non-targeted metabolomic analysis using liquid chromatography coupled with mass spectrometry (LC-MS) on plasma samples taken from 380 asthmatic children belonging to the “Genetic Epidemiology of Asthma in Costa Rica” cohort. The study identified specific metabolites associated with three clinical characteristics related to the severity of asthma. In particular, bronchial hyperresponsiveness (AHR) was correlated with 91 of the 574 profiled metabolites, while the FEV1/FVC ratio pre- and post-bronchodilator was associated with 102 and 155 metabolites, respectively. The results suggest the existence of a metabolomic fingerprint associated with the severity of the disease and, at the same time, highlight discrepancies between the metabolic profiles related to the different clinical traits, emphasizing the need for further investigation for a more accurate classification of the disease [35]. In a subsequent study, Kelly et al. [36] pursued the objective of validating metabolomics-based asthma endotypes, integrating the data generated by the 2017 study with longitudinal phenotypic data obtained from a well-characterized independent cohort, part of the Childhood Asthma Management Program (CAMP) [37]. The analysis was conducted on a common set of 589 metabolites, subsequently reduced to 398 molecules confirmed at level 1, according to the Metabolomics Standards Initiative criteria. By applying advanced computational approaches, such as Similarity Network Fusion (SNF) and spectral clustering, the authors identified five distinct asthma metabo-endotypes, each associated with differential phenotypic characteristics, including pre- and post-bronchodilator FEV1/FVC values. The reproducibility of the metabo-endotypes was also confirmed in the validation cohort. The main discriminating metabolites included cholesterol esters, triglycerides and fatty acids. These results confirm the relevance of metabolomics as a useful tool for the identification and validation of clinically significant endotypes of pediatric asthma [36]. Another metabolomic study on plasma was conducted by Fitzpatrick et al. [38]. The study involved 494 children between the ages of 6 and 17 (257 normal weight, 99 overweight and 138 obese) and the objective was to confirm that obesity is a crucial factor in complicating the clinical manifestations of pediatric asthma. The outcomes evaluated were asthma control, quality of life, lung function and exacerbations, together with responsiveness to the administration of systemic corticosteroids to which a subgroup of patients was subjected. The results showed that obesity was responsible for greater symptoms, a worse quality of life and a greater number of exacerbations, persisting for a year despite a greater intake of pharmacological therapy. Treatment with intramuscular triamcinolone also resulted in only a minimal clinical improvement in asthma control and lung function in obese children. Specific markers of systemic inflammation were also identified in obese children. Indeed, the values of leptin, C-reactive protein and some amino acid metabolites associated with glutathione synthesis and oxidative stress were altered in the obese asthmatic group. Again, within the same group, in the presence of uncontrolled asthma, the concentrations of metabolites linked to the arginine pathway were lower than in the group with controlled asthma at 12 months [38].

As far as metabolomics studies on the serum of asthmatic children are concerned, the first study conducted on the subject was by Tobias et al. [39]. The study focused on the association between nutritional status, lung function and metabolic markers in obese asthmatic children, correlating serum carotenoid and fatty acid levels with lung function indices and with insulin resistance and dyslipidemia, in a cohort of adolescents (4 groups, 39 asthmatics obese, 39 asthmatics normal weight, 38 obese controls and 42 controls normal weight). Obese asthmatic children had lower total carotenoid values, results that positively correlated with the predicted FEV1 percentage (in obese asthmatics) and inversely correlated with insulin resistance. Different values of the ratio of n-6/n-3 polyunsaturated fatty acids (PUFAs) were found in the same group, which were inversely correlated with the percentage of predicted FEV1 (in obese asthmatics) [39]. A few years later, the study by Thompson et al. [40] tried to define the obese pediatric asthmatic endotype more clearly, analyzing only asthmatic children, half of whom were of normal weight and the other half obese. They conducted a multi-omic analysis with SNF and mediation analysis on five sets of predictive data, including anthropometric measurements, metabolic profile, level of certain nutrients, transcriptome of Th cells and DNA methylome. This approach allows the quantification of both the individual and composite contribution of the predictive datasets to the obese asthma phenotype. The assessment was based on several parameters: lung function scores (spirometry and lung volume quantification using nitrogen washout), the Composity Asthma Severity Index (CASI), which assesses the severity of the asthmatic condition, and the Asthma Control Test (ACT) to asses the level of disease control. After testing 21 fusions for the 21 different combinations of the 5 predictive datasets, it was found that among the 7 of the 21 combinations that were capable of predicting 5 or more lung function indices, the samples in the 2 clusters were identical for 6 combinations and included a subset of participants, specifically 54 of the 89 total samples, for which data were available on all variables in all datasets (120 patients initially enrolled). Cluster 2, composed of 26 subjects, had lower lung function indices, higher inspiratory capacity, greater trunk and peripheral body fat, metabolic abnormalities including insulin resistance, high leptin levels and low adiponectin levels, alterations in the nutrient profile including lower levels of carotenoids and n6 PUFA, together with immune disturbances. Analysis of the results showed that these alterations were predictive of the obese asthmatic phenotype both individually and interdependently, with the most significant effect associated with the measurement values of trunk adiposity, outlining a pivotal role of this adiposity in the endotyping of childhood asthma associated obesity. However, none of these alterations proved to be predictive in either the CASI or ACT classification, the latter reflecting symptom-based assessment [40]. A further study aimed at understanding the relationship between asthmatic endotypes and nutritional status was conducted by Qu H.Q et al. [41]. In a large cohort of asthmatic children (602) and their respective controls, a metabolomic analysis with NMR was applied. Results showed reduced levels of citrate, ketone bodies, and two amino acids (histidine and glutamine) in asthmatics compared with controls. In contrast, lipid metabolites lost significance after adjusting the data to exclude the influence of obesity, except for the ratio of free cholesterol to total lipids in mean very low-density lipoprotein (VLDL) and the percentage of the ratio of saturated fatty acids to total fatty acids [41]. Other serum-level investigations were conducted by the group of Chiu et al., who wanted to investigate, through a multi-omic approach (metabolomics and microbiomics), the presence of possible host–microbiota interactions and the various asthmatic endotypes [21,42]. They first conducted a metabolomic analysis of the serum, together with shotgun sequencing of the airway microbiome, in asthmatic children sensitized to dust mites and healthy controls. The results demonstrated the presence of specific airway microbial species linked to changes in circulating metabolites and IgE responses of patients. These observations seem to suggest a strong association between respiratory microbes and disturbed circulating metabolites in pediatric mite-sensitized asthma, revealing possible etiologic but also diagnostic implications. Specifically, a positive correlation emerged between Prevotella sp. oral taxon 306 and dimethylglycine, both of which were reduced in patients, supporting a hypoxic environment linked to pediatric asthma sensitized to mites, as this metabolite is correlated with oxygen uptake by tissues and a lower accumulation of lactate [42]. Subsequently, a metabolomic analysis of the serum and a 16S rRNA sequencing of the intestinal microbiota were conducted on 3 different cohorts of children, including 15 children with poorly sensitized non-atopic asthma, 13 with highly sensitized atopic asthma and 25 healthy controls [21] together with an integrative analysis to assess the presence of associations between the two asthmatic endotypes and allergen-specific IgE levels. The results showed the presence of four metabolites (tyrosine, isovalerate, glycine and histidine) specific for low-sensitized asthma and one metabolite (acetic acid) strongly correlated with highly sensitized asthma. Integrative microbiome analysis also revealed a strong association between acetic acid, characteristic of highly sensitized patients, and airway microbiota, supporting the presence of strong host–microbe associations and asthmatic endotypes [21]. The analysis of the various metabolomic studies on blood samples (plasma and serum) has highlighted an important heterogeneity, probably mainly justified by the metabolic complexity of pediatric asthma but also by the use of different analytical techniques and distinct enrollment criteria. Despite this, there is a recurrent presence of alterations in some specific pathways such as arginine, fatty acids and oxidative stress, which could therefore represent the basis for a detailed endotyping.

Feces have also been used as a matrix for the study of asthmatic endotypes. Lee-Sarwar et al. [43] analyzed the fecal metabolome and microbiome in 3-year-old asthmatic children. The frequency of wheezing was also assessed through questionnaires. The results allowed the identification of specific microbial taxa and metabolites associated with the frequency of wheezing. Specifically, the genus *Veillonella* and the metabolites of the histidine pathway characterized subjects with a high percentage of wheezing [43]. Fecal and plasma metabolomic data were studied by Gomez-Llorente et al. [44]. The work focused on the study of obesity-related asthma, through a multi-omic analysis aimed at characterizing the clinical phenotype of allergic asthma, especially in obese children. Clinical data, plasma and fecal inflammatory biomarkers, metagenomics and metabolomics were combined in a cohort of 46 asthmatic children between 4 and 13 years of age (only inhaled corticosteroid treatments were allowed), of whom 13 were of normal weight, 8 were overweight and 25 were obese. The results of the study revealed higher leptin levels and lower plasma acetate concentrations in the obese allergic asthmatic phenotype. Variations in the fecal metabolome were observed in children with worse asthma outcomes, higher levels of fecal D-lactate and D/L lactate ratio, together with a higher relative proportion of plasma creatinine. On the other hand, lower levels of plasma citrate and dimethylsulfone characterized children with persistent asthma. Changes in the microbiota were also observed, with alterations in the *Clostridiales* (lower values) in obese children and an unclassified member of the RF39 order belonging to the *Mollicutes* class (higher values) in children with the worst asthmatic outcomes. These results led the authors to confirm the extreme molecular heterogeneity of the allergic asthma phenotype and the usefulness of modern high-throughput technologies in characterizing the various underlying endotypes with greater precision, highlighting the use of omic technologies to examine the clinical phenotype at a more holistic level [44].

A single study was conducted on urine samples from children with severe asthma by Park et al. [45] to evaluate corticosteroids resistance. They analyzed the urine of 15 corticosteroid-responding and 15 non-responding asthmatic children using liquid chromatography coupled with Fourier-Transform Mass Spectrometry (FTMS). The results identified five possible biomarkers, involved in tyrosine metabolism, in the degradation of aromatic compounds and in glutathione pathway associated with corticosteroid resistance in children with severe asthma [45]. From the analysis of metabolomic studies on feces and urine, two other matrices of interest due to the low invasiveness of sampling, significant potential for study emerges regarding the impact of nutritional status and the contribution of microbiota. However, the total absence of standardized protocols significantly limits the clinical use of this data, which could be corroborated by the integration of multi-omic data.

Finally, a further limitation that vitiates the clinical application of metabolomic data in pediatric asthma by affecting most studies regardless of the sample analyzed can be found in the poor integration between metabolomic data and the known immunologic classification of endotypes. Indeed, analysis of the literature suggests that most studies stratify patients only by clinical features (such as obesity, severity, response to corticosteroids or disease control) [30,31,32,33,34,35,36,38,39,40,44,45]. This severely limits the unambiguous association of metabolic profiles with underlying immunologic mechanisms. Only a few recent studies have attempted a more direct link between metabolites and immunologic profiles, such as IgE sensitization or Th2 inflammation [21,41,42].

## 4. Pediatric SARS-CoV-2

Compared to adults, analysis of early data on SARS-CoV-2 infection in children showed asymptomatic or mild forms with a significantly lower mortality rate [46]. In fact, severe disease in pediatric age was rare, unless predisposing conditions such as age < 12 months, immunodeficiency, chronic lung disease or co-infection were present [47]. Dong Y et al. collected data from 2143 Chinese pediatric patients with COVID-19, showing that more than half had a mild disease and only less than 1% developed severe or critical symptoms [48], confirming what has also been observed in other research conducted during the same period [49,50]. A review of the literature was conducted by analyzing 1124 pediatric cases stratified by clinical severity, as follows: 14.2% asymptomatic, 36.3% with mild forms (fever, cough, gastrointestinal symptoms), 46% with pneumonia (moderate form), 2.1% with dyspnea and desaturation (severe form) and 1.2% with acute respiratory distress syndrome and/or multi-organ dysfunction (critical form) [50]. Symptoms such as anosmia and ageusia, although present, are underestimated, especially in children <3 years of age [51]. Reported skin manifestations include chilblain-like, urticarial and maculopapular lesions, but the causal link with SARS-CoV-2 is not yet well defined [52]. Respiratory symptoms in children, which are often non-specific, can overlap with those of common viral infections such as influenza, rhinovirus or RSV. The lower severity of the infection in children could be related to the way the virus enters the cells. SARS-CoV-2 uses the spike glycoprotein to bind to the angiotensin-converting enzyme 2 (ACE2) receptor, with the support of the transmembrane protease serine 2 (TMPRSS2), facilitating the entry of the virus into the host cell [53,54,55,56,57]. ACE2 expression is higher in the nose, especially in the ciliated cells, but reduced in the lungs, relatively restricted to type II alveolar cells and can be modulated by inflammation and environmental stimuli [58]. Moreover, in children, unlike adults, “double-positive” cells, i.e., those expressing both ACE2 and TMPRSS2, are rather rare. This prevents the overexpression of interleukin-6 (IL-6), which is responsible for the over-activation of the immune system, a potential cause of the cytokine storm [59,60,61,62,63]. The levels of cathepsin L/CTSL1, a protease necessary for the activation of the spike protein, are also lower [60,64]. In addition, there is more active innate immunity, with greater numbers of T and B lymphocytes and NK cells, although there remains a general immaturity of the childhood immune system, as evidenced by data on the susceptibility and severity of other viral respiratory tract infections in children [65]. There is also greater microbial colonization in childhood, a condition that may limit the spread of the virus [63]. Adaptive immunity also appears to be greater; in fact, the study by Dowell et al. [66] showed that in children, antibody responses against the spike protein are higher and there is greater seroconversion that boosted responses against seasonal beta-coronavirus, although neutralization of viral variants was comparable between children and adults. In addition, specific T-cell responses were more robust and persistent than in adults, supporting the presence of preexisting cross-reactive responses to seasonal coronaviruses responsible for effective immunological memory [66].

However, the natural evolution of RNA viruses, in which the presence of a single-stranded genome of around 30,000 nucleotides is subject to a high frequency of replication together with a poor error-correction capacity on the part of RNA polymerase, must be considered. These characteristics appear to be decisive in favoring the spontaneous accumulation of mutations. This occurs mainly in the coding region for the spike protein, which is crucial for viral entry. If these mutations confer evolutionary advantages on the virus, they can be positively selected, resulting in increased transmissibility and cell tropism, but also in a higher capacity for evasion of the immune response and in increased pathogenicity. Thus, there has been an accumulation of mutations responsible for the emergence of numerous viral variants, characterized by functional mutations in the spike protein, particularly in the receptor-binding domain (RBD), which increase the affinity for ACE2 or reduce the efficacy of neutralizing antibodies. These conditions have given rise to subsequent research comparing the different impact of variants on children [67]. In this regard, a recent review of the literature [68] showed that although Omicron has a higher transmissibility, clinical forms in children tend to be milder than those observed with earlier variants. In particular, a comparative analysis of the main studies conducted on the impact of the different SARS-CoV-2 variants in children [69,70,71] shows a higher hospitalization rate and clinical severity during the Alpha and Delta waves, with a subsequent decline in intensive care admissions during the Omicron wave. Specifically, the Alpha and Delta variants have generally been associated with more severe clinical forms, while Omicron is correlated with a milder course in most pediatric cases. However, it has been found that, very often, clinical manifestations also vary depending on the geographical context, the population studied and the concomitant circulation of other pathogens [69,70,71].

The most recent viral variants are also associated with a reduction in the incidence of MIS-C, suggesting a possible evolution of the infection towards a Kawasaki disease-like phenotype and a less aggressive immune involvement [68]. Indeed, Abhram et al. [72] recently published a retrospective study, conducted in two pediatric hospitals, which analyzed 129 cases of MIS-C that occurred during the four pandemic waves, each associated with a different variant of SARS-CoV-2 (Ancestral, Beta, Delta, Omicron). The results showed that the clinical severity and phenotype of MIS-C remained essentially stable, although the number of cases decreased progressively from 38% in the first wave (Ancestral), to 16.3% in the second (Beta), to 33.3% in the third (Delta) and finally to 12.4% in the fourth (Omicron). This led the authors to conclude that although the clinical course was generally severe and constant, the Omicron wave recorded the lowest number of hospitalizations for MIS-C, probably due to the increase in seroprevalence in the pediatric population because no patients were vaccinated at the time of diagnosis [72]. Finally, data from a very recent systematic review of the literature [73] showed that the post-COVID-19 condition (PCC) also decreased with Omicron, probably due to the generally milder clinical course associated with this variant. In fact, even in pediatric age, an association has been found between the severity of the acute disease and the incidence of PCC, with more severe cases correlating to a greater probability of developing persistent symptoms, independently of the variant involved [73].

However, independently of the natural viral evolution, an important risk factor for severe forms is obesity. Indeed, obese children, compared to normal-weight children, have shown more marked symptoms, a greater need for hospitalization in intensive care and higher mortality rates, albeit low in absolute terms [6,73,74,75,76]. This is supported by data from systematic reviews of the literature that confirm the role of obesity as an independent risk factor for severe forms and long-COVID-19 [77,78]. Concurrently, during the pandemic, an increase in weight and BMI has been observed, especially in children, linked to a sedentary lifestyle, greater use of electronic devices, dietary changes and school closures [13,14,79,80,81,82,83,84,85,86,87]. In particular, weight gain has been correlated with the duration of distance learning, similar to what was already observed during school holidays [75,80]. Nevertheless, in children already undergoing treatment for obesity or diabetes, greater family involvement and the use of telemedicine had a restraining effect on weight gain [82,88,89]. Digital platforms have proven useful in remote nutritional management [90]. Finally, the contribution of pandemic-related stress to childhood obesity should be emphasized, as it negatively influences hormonal homeostasis (reduction in leptin, increase in ghrelin), the HPA axis and the intestinal microbiota, favoring dysbiosis and insulin resistance [86,91,92].

## 5. Pediatric Asthma and SARS-CoV-2

At the beginning of the SARS-CoV-2 pandemic, pediatric asthma raised concerns about the potential risk of more severe forms of COVID-19. This is because asthma is a chronic inflammatory airway disease associated with bronchial hyperresponsiveness and viral respiratory infections are responsible for up to 80% of exacerbations of the condition [3,90]. Its severity, according to the GINA guidelines, is defined by the frequency of symptoms and the need for background therapy [93]. Moreover, the presence of exacerbations, characterized by worsening respiratory symptoms, often requires therapeutic adjustment [93]. From a pathophysiological perspective, asthma involves mucosal hypersecretion, epithelial damage and airway obstruction, associated with a delayed and ineffective innate antiviral response. Furthermore, the use of inhaled corticosteroids, a cornerstone of asthma treatment, was initially considered a possible risk factor, as it may delay viral clearance and induce local immunosuppression [6,18,94,95,96]. These factors have led to the hypothesis that asthmatic patients may be more vulnerable to SARS-CoV-2 infection and its complications [96,97,98]. Indeed, early observations in the United States, which identified asthma as a frequent comorbidity in adult patients hospitalized for COVID-19, seemed to confirm this concern [98]. However, subsequent research has contradicted this hypothesis. Indeed, some studies have found a reduction in hospitalizations for asthma exacerbations during the pandemic; however, this can probably be primarily attributed to the reduced circulation of respiratory viruses due to hygiene and sanitary measures and the lockdown [19,99]. One of the first systematic reviews on the subject, conducted by Castro-Rodriguez et al. [99], excluded asthma as a risk factor for infection or its severity, although it highlighted the need for further investigation. With regard to the specific pediatric field, the multinational study Pediatric Asthma in Real Life (PeARL) [8] analyzed 1054 asthmatic and 505 non-asthmatic children, aged between 4 and 18 years, during the first wave of the pandemic, comparing the frequency of respiratory and febrile symptoms with data from the previous year. The results showed an improvement in asthma control in children during the pandemic period, attributed to less exposure to environmental triggers and greater adherence to therapy [8]. This evidence was confirmed by a systematic review by Yang et al. [100], which reported a significant improvement in asthma control in the pediatric population compared to the pre-pandemic period. Another systematic review [101] investigated the factors that can influence the susceptibility of the pediatric asthmatic population to COVID-19, such as asthmatic phenotypes, the use of inhaled corticosteroids and the impact of restrictions. It has been shown that some immunological characteristics of asthmatic children, including reduced ACE2 receptor expression and increased eosinophils, may act as protective factors, together with the indirect benefits of containment measures and increased therapeutic compliance [101].

From a pathophysiological point of view, the further reduced expression of ACE2 in the airways, found above all in children with allergic or atopic asthma, has been proposed as a possible protective factor [44]. Indeed, ACE2 expression, induced by IFN-I, is lower in subjects with high allergic sensitization, as demonstrated by Jackson et al. in three pediatric and adult cohorts [3].

This regulation would be attributed to type 2 cytokines, in particular IL-13, which appears to suppress ACE2 while increasing TMPRSS2 [44,102,103,104]. However, the effect on TMPRSS2, although confirmed by studies conducted on bronchial biopsies of asthmatic patients [105], would be largely compensated by the concomitant reduction in ACE2 [3,98]. It has also been hypothesized that the action of IL-13 reduces intracellular viral load and intercellular transmission, increasing the protection of the respiratory epithelium and limiting viral replication [44]. The state of IFN-I deficiency, typical of some forms of asthma, could also be protective against the cytokine storm induced by SARS-CoV-2, contributing to less aggressive pulmonary inflammation [99].

The data available to date show that although pediatric asthma may represent a potential risk factor for severe COVID-19, as a condition subject to virus-induced exacerbations, it cannot be consistently identified as a significant comorbidity. However, questions arise in this regard, and it is important to focus on the causes; that is, whether these results were caused by the blocking measures and/or the higher prevalence of pediatric eosinophilic asthma. In addition, it would be useful to consider whether stratifying the impact of infection according to different asthma endotypes would have produced different results. Regarding the correlation between asthma endotypes and COVID-19, a possible protective effect has been hypothesized in the presence of atopic/allergic asthma (Th2-high) to be attributable to the peculiar features of atopy, which is the genetic predisposition to produce a type 2 (Th2) immune response upon exposure to environmental antigen. Indeed, allergic sensitization has been shown to correlate with decreased levels of ACE2, elevated levels of total immunoglobulin E (IgE) and type 2 inflammatory cytokines such IL-4, IL-13, and IL-5 released by cells of the innate and adaptive immune systems. These cytokines are responsible for both the inflammatory process typical of asthma and airway remodeling, together with aberrant viral sensing and altered intracellular signaling, which result in deficient interferon responses to viral infections [44,103,105,106,107]. These asthmatic patients are also characterized by sensitization to aeroallergens responsible for high levels of total immunoglobulin E and elevated eosinophil counts. This asthmatic endotype often originates in childhood and is frequently associated with other atopic diseases such as allergic rhinitis and dermatitis (AD) [3,98]. Its incidence, very common in the pediatric population, reaches a peak in early childhood and decreases steadily with age, unlike the non-allergic phenotype which has a low prevalence in childhood and peaks in late adulthood [106]. Nevertheless, analysis of the literature shows non-univocal results. In fact, the study by Muñoz et al. [108], which mainly included patients with non-allergic asthma, did not show a higher incidence or severity of COVID-19 in this population. In this case, the absence of an association between eosinophilia and protection from the virus contrasts with the findings of Ferastraoaru et al. [109], who instead observed a protective effect of pre-existing eosinophilia (ESR ≥150 cells/μL) with respect to hospitalization and mortality from COVID-19. Finally, in the study by Muñoz et al. [108], no clear relationship was found between the dose of inhaled corticosteroids and the severity of COVID-19, while other research, both in vivo and in vitro, has shown that these drugs can suppress viral replication and cytokine production [105,106,110]. Furthermore, Peters et al. [111] have shown that the use of inhaled corticosteroids is associated with a reduced expression of both ACE2 and TMPRSS2 in asthmatic patients.

The impact of different SARS-CoV-2 variants on pediatric asthma exacerbations has also been investigated. In fact, Gaietto et al. [11] conducted the first large-scale individual retrospective analysis of 573 asthmatic children with COVID-19 to study the incidence of asthma exacerbations associated with SARS-CoV-2 infection during the three major pandemic waves: Pre-Delta (July 2020–June 2021), Delta (August–December 2021) and Omicron (December 2021–August 2022). Results obtained through multivariate logistic regression showed that the rate of asthma exacerbations was significantly higher during the Omicron wave (40.2%) than during Delta (26.2%) and Pre-Delta (22.6%) (*p* = 0.002). However, although after adjustment for known confounding variables such as age, gender, race, BMI, income, emergency department presentation and asthma severity, the probability remained significantly increased, it was no longer statistically significant once adjusted for asthma control. This result suggests that sub-optimal symptom control may be a contributing factor in susceptibility to exacerbations, regardless of viral variant. Nevertheless, from a clinical point of view, the Omicron wave was associated with a higher frequency of exacerbations. According to the authors, this could be due both to a worsening of asthma control in the affected population and to the specific virological characteristics of the Omicron variant. Specifically, a greater predilection for the upper airways, a lower affinity for TMPRSS2 together with a reduced capacity for cell fusion. This is compounded by the relaxation of containment measures (e.g., masks, distancing) and the subsequent reappearance of other co-circulating respiratory viruses that may have contributed to increased bronchial reactivity [11]. Similar results were obtained in the study by Chan et al. [12], who analyzed 18,932 children affected by COVID-19 during the Alpha, Delta, Omicron BA.1 and Omicron BA.2 waves. It emerged that children with asthma were less represented during the Alpha and Delta waves, while they were significantly more represented during the Omicron BA.2 wave. In contrast, the clinical severity between children with and without asthma did not differ significantly, but the duration of hospitalization was slightly longer in asthmatic patients, particularly during Delta and Omicron BA 2. Again, the results seem to suggest a possible interaction between the circulating viral variant and the susceptibility of asthmatic children, particularly in the forms with greater upper airway tropism [12].

## 6. Shared Inflammatory and Metabolic Networks Between Asthma and Pediatric SARS-CoV-2

The review of the literature showed that most cases of COVID-19 in children are mild. However, it should be emphasized that cases of critical and fatal infection are not negligible, often related to specific clinical characteristics. These characteristics therefore allow for a risk stratification for young people at risk of severe COVID-19, useful in guiding personalized treatment [7,77]. This is possible through a detailed analysis of the main specific risk factors for the pediatric population and the evaluation of the main shared metabolic pathways, in an attempt to optimize both therapeutic and preventive strategies. In fact, from the outset, an initial application of systems biology, through the integration of modern high-throughput technologies, had identified some metabolic pathways shared between COVID-19 and the main comorbidities (diabetes, hypertension, cardiovascular diseases, chronic kidney diseases and cancer). This immediately drew attention to the need for highly personalized treatments for people with COVID-19 and other pathological conditions [13]. Nevertheless, most studies aimed at developing new therapeutic interventions have been undertaken in the adult population [7].

In childhood, the most common chronic pathological condition is asthma, but it is also important to consider the constant increase in the prevalence of childhood obesity, which is also emerging as one of the most common comorbidities of difficult-to-treat asthma [28]. Indeed, in order to undertake successful treatment of pediatric asthma, a careful analysis of its complex and varied molecular variants is essential. This makes it necessary to focus on the presence of different endotypes, which can describe the distinct pathophysiological mechanisms at the cellular and molecular level of each asthmatic phenotype. For example, a growing body of scientific evidence suggests that the asthmatic endotype associated with obesity represents a biologically distinct condition [29]. Therefore, it may be advantageous for the endotyping of young asthmatic patients, often based mainly on clinical characteristics and laboratory parameters, to be improved. Specifically, it could be deepened with unbiased molecular tests but also expanded through new high-throughput technologies. In this regard, omic approaches, including metabolomics and microbiomics, with minimally invasive sampling, have so far provided precise and impartial evidence to support existing endotypes but have also suggested the conceptualization of new endotypes, together with the identification of biomarkers capable of predicting response to treatment and disease progression [16,21,22,23,24]. In fact, it has been discussed how, to date, there are now numerous pediatric metabolomic studies supporting the presence of key metabolites associated with disease severity, inflammatory response and clinical control. Specifically, carnitines, creatinine, thiamine, saturated fatty acids, some aromatic amino acids and carboxylic acids showed different patterns in obese asthmatic children compared to normal weight children.

With specific regard to asthma in the presence of COVID-19 infection, in order to highlight precise targets for intervention, a population study was conducted based on network medicine for the investigation and validation of pathological manifestations and the design of specific drugs, through integrative analysis of metabolomic and transcriptomic data (bulk and single-cell) [23]. This research, through the analysis of differential metabolites, has highlighted the presence of reduced levels of L-arginine and L-citrulline in patients infected with SARS-CoV-2, in analogy with what has been shown in asthmatic patients. Higher levels of these metabolites have in fact been shown to protect against asthma [23,41]. Furthermore, metabolomic endotyping of asthmatic children has also revealed critical issues related to the arginine pathways correlated with exacerbations of the disease, especially in obese children. In this subgroup, alterations in arginine metabolism are closely related to nitric oxide dysfunction and worsening lung function, suggesting the opportunity for targeted interventions on this pathway in the context of co-infection or severe uncontrolled asthma [31,41].

It has also emerged that COVID-19 shares an intermediate inflammatory molecular profile with asthma. Specifically, it has been observed that SARS-CoV-2 infection has increased the expression of several key inflammatory genes, including IRAK3, a gene for asthma susceptibility, and ADRB2, a genetic factor that regulates the risk of onset and severity of asthma, resulting in an increased risk and severity of this disease. Correlations have also emerged with NF-κB inhibitor alpha (NFKBIA), a gene responsible for critical transcriptional responses in childhood asthma [23]. Also in this context, metabolomics studies aimed at endotyping pediatric asthma have revealed a high inflammatory state both systemic [41] and intestinal [42]. These alterations are accompanied by variations in the profile of fecal and plasma metabolites, such as an increase in lactate, a decrease in citrate, dimethylsulfone, short-chain fatty acids and amino acid modifications, reflecting a state of chronic inflammation and dysbiosis associated with endotypes linked to increased disease severity [29,41,42].

All this is in agreement with the results of specific research on the pediatric population, conducted by Gao XM et al. [112], to study the pathogenesis of childhood asthma by examining the genetic components involved in this disease. They applied a new approach to predict which genes that are still unknown may interact with the known pathogenic genes of asthma, with the aim of identifying other potential genes involved in the pathogenesis. Two groups were created: network A with seed genes, i.e., known identified genes, and network B, with potentially pathogenic genes that interacted with at least two genes from network A. The biological pathways of the seed genes and candidate genes as potentially pathogenic were then analyzed based on the information obtained from the KEGG database. The results showed the presence of three biological enrichment pathways shared between the seed genes and the candidate genes, and the NFKBIA and BIRC3 genes, belonging to group B, shared all three biological pathways. This led the authors to conclude that NFKBIA and BIRC3 may be pathogenic genes [112]. The important role of NFKBIA in the regulation of transcriptional responses to the main childhood lung diseases, including asthma, also emerged in the work of Ali et al. [113]. They investigated the functional and clinical impact of human genetic variants in the promoter of NFKBIA, which encodes IκBα, the main negative regulator of NF-κB, highlighting how NFKBIA/IκBα is a central hub in the transcriptional responses of the main childhood lung diseases. The authors therefore concluded that genetic variants in the NFKBIA promoter influence the expression of the NFKBIA gene, the expression of the IκBα protein and the inflammatory responses mediated by toll-like receptors (TLRs), revealing critical aspects in the genetic predisposition to childhood asthma [113]. The involvement of TLR signaling pathways supports the complex molecular network that correlates SARS-CoV-2 infection and the main related risk factors. In fact, this signaling pathway, which in SARS-CoV-2 infection is the main responsible for the secretion of pro-inflammatory cytokines and cytokine storm, may represent part of the intermediate inflammatory molecular profile shared between COVID-19 and asthma, but also highlights the involvement of other relevant comorbidities, such as diabetes and obesity [23,113]. Recent studies have also highlighted the interaction between TLRs and lipid metabolism, suggesting a potential link between immune activation, metabolic dysfunction and the risk of chronic inflammation in contexts such as severe asthma and COVID-19 [114]. Indeed, it seems that NF-κB and the TLR signaling pathways represent an important link between the impairment of insulin resistance and obesity-induced inflammation [114].

The inflammatory molecular pathways of IL-6 could also suggest an important correlation between COVID-19 and the main pediatric comorbidities, such as asthma and obesity. In fact, both meta-analyses and systematic literature reviews have confirmed the association between significantly elevated IL-6 levels and adverse clinical outcomes in COVID-19 patients [32,115]. Increased IL-6 levels are also correlated with increased BMI in children and an association has emerged between IL-6 and the likelihood of asthma exacerbation [26,28]. It is indeed known that excess adipose tissue leads to an overproduction of pro-inflammatory leptin, which in turn feeds the inflammatory process and is responsible for the synthesis of pro-inflammatory cytokines such as IL-6, which can at the same contribute to the pathogenesis of asthma [116]. All these metabolic alterations are confirmed by the review on the use of metabolomics in the subtyping of childhood obesity-related asthma published by Makrinioti et al. [29]. In fact, the authors highlight how a greater insulin resistance characterizes obese asthmatic children and among these, those with the worst control of the pathology have lower plasma levels of long chain n3 polyunsaturated fatty acids [29]. This reflects what was previously discussed regarding metabolomic endotyping of the obese asthmatic phenotype, which revealed decreased carotenoids, n-3 PUFAs and vitamin B6, as well as increased lactate, sphingolipids and histidine metabolites, all markers associated with immunometabolic dysfunction and worse lung function [29,31,42].

It therefore appears necessary to undertake deeper investigations of the molecular characteristics of the pediatric asthmatic phenotypes, especially the obesity-related one, consisting of different endotypes, with the aim of undertaking personalized treatments and effective preventive strategies even in the presence of potentially serious infections such as COVID-19. Unfortunately, to date, there are still many gaps in our knowledge of the pediatric obesity-related endotype and the association between asthma and obesity is still a matter of debate, probably due to the underlying complex multifactorial relationship that casts doubt on the direct causality between these two entities [21,28]. On the one hand, recent systematic reviews of the literature seem to suggest that obesity represents an important risk factor for asthmatic pathology [21,114], adding to the evidence regarding its contribution in terms of the severity of the pathology. In fact, an increase in asthmatic symptoms has been observed not only in terms of prevalence but also of severity in children when associated with excess weight [26,28,115]. Furthermore, although severe asthma represents a particular and rare endotype, obese asthmatic patients are at greater risk of developing it, requiring significant pharmacological treatments [13,25]. On the other hand, other research supports the possibility that asthma itself contributes to the increase in obesity, especially if it is not well controlled [27,28]. In this regard, Chen and colleagues [27] studied the effects of asthma and related phenotypes on the development of obesity in a cohort of non-obese children. They examined the incidence of obesity during a 10-year follow-up to evaluate the hypothesis that children who have asthma in the first years of life are at greater risk of developing obesity during childhood and adolescence. The results showed that non-obese children with asthma at baseline had a 51% higher probability of developing obesity during follow-up compared to children without asthma at baseline. In addition, patients who used asthma rescue medication at the start of the study had a significantly lower risk of becoming obese during follow-up compared to participants who did not use the medication. These results suggest that the use of asthma rescue medication in early childhood could protect against weight gain in later life, with a view to a preventive approach to obesity through early diagnosis and treatment of childhood asthma [27].

## 7. From Asthma Endotypes to SARS-CoV-2 Risk Stratification: Integrative Analysis

In recent years, the clinical profile of pediatric SARS-CoV-2 infection has undergone major changes, mainly due to the emergence of new viral variants. Whereas in the early phase of the pandemic children were often paucisymptomatic or asymptomatic, the new variants have led to an increase in clinical manifestations, hospitalizations and post-viral complications [117,118]. This development fully reflects the dynamic nature of the viral agent and the importance of considering, according to the epidemiological triad model, the interaction between agent (virus), host (asthma patient) and environment (exposure, containment measures) [119]. In this regard, it is important to note that children’s adherence to containment measures may significantly differ from that of adults and this behavioral dimension, frequently underestimated, represents an additional environmental variable that can influence exposure risk and clinical outcomes in vulnerable pediatric populations [120].

The inclusion of these aspects in the risk stratification process therefore allows a more realistic interpretation of clinical variability not only for predictive but also for therapeutic purposes. The importance of an integrated approach to stratification, albeit referring to the adult population, was highlighted already during the early stages of the pandemic. Indeed, Ssentongo et al. [121], through supervised ML methods, classified patients into five clinical subgroups based on models that integrated comorbidities, demographic characteristics and laboratory parameters, revealing how known risk factors (including asthma and diabetes) were not uniformly associated with specific clinical pictures. Neither was viral genotype predictive of a specific course, further emphasizing the need for more complex stratification models [121].

**Figure 1:**
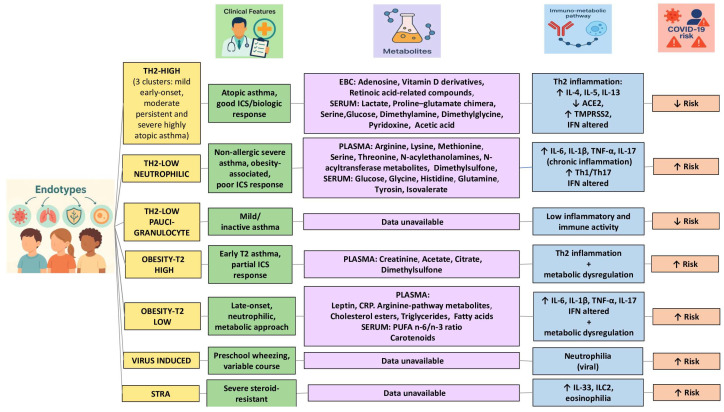
Clinical, metabolomic and immunologic features of pediatric asthma endotypes in relation to COVID-19 susceptibility. Abbreviations: EBC—exhaled breath condensate; ↓—decreased; ↑—increased; Th2—T helper type 2; ICS—inhaled corticosteroids; ACE2—angiotensin-converting enzyme 2; TMPRSS2—transmembrane protease, serine 2; Th17—T helper type 17 cells; IFN—interferon; IL-6—interleukin 6; IL-33—interleukin 33; ILC2—Type 2 innate lymphoid cells; PUFA—polyunsaturated fatty acids.

Therefore, the need for an endotype-specific approach for asthmatic patients is now clear even in the pediatric setting, especially in the presence of comorbidities such as obesity, in order to guide innovative and personalized prevention, monitoring and treatment strategies. Indeed, it is possible to hypothesize different vulnerability depending on immunologic characteristics, metabolomic profile and therapeutic response, as schematized in Figure 1. Only metabolites clearly identified in metabolomic endotyping studies and associated with specific pediatric asthma endotypes, either immunologically defined or inferred on the basis of clinical and biological features, were included [21,30,32,33,34,36,38,39,40,41,42,44]. The implementation of this area of research can take place with the identification of specific discriminatory molecular biomarkers (metabolites), validated in large cohorts, with a view to their use in precision medicine.

Thus, in the contest of clinical risk stratification, endotypes characterized by a high Th2 response present eosinophilic inflammation mediated by IL-4, IL-5 and IL-13, conditions that are associated with a reduced expression of ACE2 on respiratory epithelial cells. This condition could be the main factor responsible for a relative protection against COVID-19 infection [122]. This protection may be further favored by the immune system typical of atopic asthma, which is less prone to hyperactivated Th1-type inflammatory responses. This immune arrangement seems to effectively compensate the deficient interferon response [123].

A different picture occurs in low Th2 endotypes, including obesity-related endotypes when associated with a neutrophilic, which appear to be characterized by high levels of IL-6, IL-1β and TNF-α and greater involvement of Th17 cells. Indeed, it is precisely these immune conditions that are responsible for both a deficient type I and type III IFN response and the persistent systemic inflammation. It emerges that reduced interferon activity (type I and III) can have opposite effects depending on the inflammatory context of the host. Thus, in subjects with a Th2-low endotype, often characterized by chronic neutrophilic inflammation, obesity or severe nonallergic asthma, interferon deficiency itself impairs the innate antiviral response, becoming a crucial factor in susceptibility to severe forms of COVID-19. In fact, this immune set-up is associated with increased viral replication, delayed immune response and increased production of pro-inflammatory cytokines such as IL-6 and TNF-α, which contribute to the worsening of clinical outcomes and the risk of severe forms of COVID-19 by increasing and prolonging the systemic inflammatory state [20,123,124,125]. In addition, there is the activation of Th1 and Th17 axes, which in a condition of reduced interferon type I and III expression and systemic inflammation may increase the vulnerability of individuals to develop more frequent PCCs, with symptoms persisting for more than four weeks after acute infection, including dyspnea, chronic cough, asthenia, sleep disturbances, and worsening asthma control. Furthermore, in the presence of neutrophilia, severe asthma, obesity and poor steroid responsiveness, the presence of chronic inflammation mediated by Th1 and Th17 cells promotes the production of IFN-γ and IL-17. This inflammatory profile, together with the persistence of an altered metabolic condition, may further increase the likelihood of long-term complications, such as recurrent exacerbations, reduced lung function, and chronic obstructive pulmonary disease-associated neurovegetative disorders [20,126,127]. In contrast, in the presence of paucigranulocytosis, with mild or nonactive asthma and low immune activation, the clinical risk is generally considered low, as it lacks significant inflammatory markers [128].

The STRA endotype, with persistent eosinophilia, ILC2 activation and steroid resistance, could also be a high-risk profile. This is mainly due to a dysregulated immune response, difficulty in pharmacological control and a high baseline inflammatory burden, all conditions capable of amplifying the long-term effects of the viral infection [128].

Finally, the viral-induced endotype, typical of preschool children with episodic wheezing, presents a variable risk, which depends mainly on the presence of an active viral infection but also on the maturity of the IFN response and on the possible concomitant respiratory or intestinal dysbiosis [129].

## 8. Conclusions

The scientific evidence concerning the presence of a shared inflammatory–metabolic axis between pediatric asthma, the most common chronic disease in childhood, SARS-CoV-2 infection and obesity, one of the main pediatric comorbidities, is mounting. It therefore no longer seems possible to adopt a uniform therapeutic approach in the context of extreme heterogeneity of asthmatic pathology, especially in children, without taking into account not only the possible presence of comorbidities, which are also closely related to the different pathological endotypes, but also the occurrence of viral infections, such as COVID-19. This requires scientific research to implement studies in this field with the aim of identifying new molecular targets and biomarkers, thus directing therapeutic choices towards precision medicine. In consideration of these findings, the role of diet, and consequently the microbiota, in modulating these shared metabolic pathways has also become a subject of study. Consequently, their impact on disease risk must be investigated when designing a personalized treatment for each patient. To date, among omics technologies, metabolomics has proven to be a promising approach for the endotypic characterization of pediatric asthma. However, its clinical applicability still remains limited.

This is mainly due to the high inter-individual variability and the lack of standardized protocols for sample collection and analysis, together with a significant complexity of data interpretation, further exacerbated by the heterogeneity of analytical platforms and data processing pipelines. These issues are the principal obstacle to standardization and the greatest limitation to clinical translation of metabolomic signatures, partly due to the absence of reliable and practical diagnostic tools for routine use.

Furthermore, most of the studies conducted involve relatively small sample sizes, which limit the statistical power and generalizability of the findings. These studies are often cross-sectional, lack longitudinal validation and show poor integration between metabolomic data and the known immunological classification of endotypes. Despite that, the first pilot studies have demonstrated the feasibility of using non-invasive samples, such as EBC and urine, to discriminate between asthma endotypes and to accurately predict the severity of the disease. These approaches could become valuable tools in the context of risk stratification in case of epidemic infections, such as COVID-19, especially if integrated into multi-omic platforms.

From a clinical perspective, integrating metabolic signatures into medical practice could enable early risk identification, guide targeted therapy selection and support personalized monitoring. This appears even more relevant in the context of future pandemics, where specific metabolic biomarkers could enable early risk stratification and the implementation of appropriate and timely therapies in asthmatic children, especially in high-risk cases with comorbidities. However, this makes it necessary to develop accessible diagnostic tools, define pediatric reference values and include validated omic biomarkers in future asthma guidelines.

## Figures and Tables

**Table 1:** Clinical–immunological features of the main pediatric asthma endotypes.

Endotype	Immunological Features	Clinical Phenotype	Treatment Response
Th2-high	↑ IL-4, IL-5, IL-13, eosinophilia, ↑ IgE, ↓ IFN-I/III	Cluster 1: mild early-onset atopic asthma (low exacerbations, preserved lung function) Cluster 2: moderate persistent atopic asthma (↓ lung function, severe hyperreactivity) Cluster 3: severe highly atopic asthma (significant symptoms, intensive therapy required, impaired lung function)	Good response to ICS and biologics
Th2-low neutrophilic	↑ Th17, IL-17, IL-12, TNF-α, neutrophilia, ↓ IFN-I/III, ↑ IFN-γ, Th1/Th17 activation	Mild early-onset non-atopic asthma (normal lung function) Severe persistent non-allergic asthma (poor control, obesity-associated, high exacerbations)	Poor response to ICS requires specialist evaluation
Th2-low paucigranulocytic	Absence of eosinophils or neutrophils, low systemic inflammation	Mild or inactive asthma, airway remodeling and bronchial hyperreactivity, persistent airflow limitation	Variable
Obesity-related (T2-high)	Eosinophilic inflammation, high BMI, predominant Th2 profile, possible ↓ IFN-I/III	Early-onset asthma with T2 inflammation	Partial, nutritional intervention may beneficial
Obesity-related (T2-low)	Neutrophilic inflammation, ↑ IL-6, IL-1β, ↑ IL-17, ↑ IFN-γ, ↓ IFN-I/III, Th1/Th17 activation	Late-onset asthma with systemic inflammation	Poor, targeted metabolic interventions required
Virus-induced	Neutrophilia (during viral infection), immature IFN-I/III responses in children, impaired antiviral defense	Preschool episodic wheeze	Variable, does not always progress to asthma
STRA	↑ IL-33, ILC2, persistent eosinophilia, sustained Th2 profile, immune dysregulation, ↓ IFN-I/III	Severe, corticosteroid-resistant asthma	Biologics needed, intensive management, poor ICS response due to steroid resistance

Abbreviations: ↑—increased; ↓—decreased; TNF-α—tumor necrosis factor-alpha; IL-33/4/5/12/13/17—interleukin 33/4/5/12/13/17; ILC2—type 2 innate lymphoid cells; ICS—inhaled corticosteroids; STRA—severe therapy-resistant asthma.

**Table 2:** Asthma endotyping metabolomics studies on EBC.

Samples	Patients	Technique	Key Findings	Clinical Relevance
EBC [30]	42 asthmatic children (8–17 years): 31 with non severe and 11 with severe asthma	LC-MS	EBC metabolomic profiling separated severe, non-severe asthma and controls Key metabolites: retinoic acid, adenosine and vitamin D derivatives	Non-invasive breathomics profiling supports asthma phenotyping and tailored therapy
EBC [31]	89 asthmatic children and 20 controls	NMR + ML	Three distinct asthma clusters emerged, differing in eosinophil levels, exacerbation rates, and family history.	ML-assisted breathomics may non-invasively reveal pediatric asthma endotypes

Abbreviations: EBC—exhaled breath condensate; NMR—nuclear magnetic resonance; ML—machine learning; LC-MS—liquid chromatography–mass spectrometry.

**Table 3:** Asthma endotyping metabolomics studies on plasma and serum.

Samples	Patients	Technique	Key Findings	Clinical Relevance
PLASMA [32]	215 asthmatic subjects, including 41 with exacerbative asthma	UHPLC-MS	32 unique cohort-independent metabolites distinguished exacerbation-prone from non-prone asthmatic children Arginine, lysine and methionine pathways the most affected	Plasma metabolomics distinguishes exacerbation-prone asthma despite high-dose ICS
PLASMA [33]	22 children with mild-to-moderate asthma (8 normal weight, 7 overweight and 7 obese) and 35 with severe refractory asthma (15 normal weight, 9 overweight and 11 obese) (9–17 years)	LC-MS	Severe and mild-to-moderate asthma showed distinct plasma metabolomic profiles involving glycine/serine/threonine metabolism and N-acylethanolamine signaling, both linked to oxidative stress	Oxidative stress–linked metabolic changes may explain corticosteroid insensitivity in severe pediatric asthma and suggest new therapeutic targets
PLASMA [34]	64 asthmatic subjects (5–12 years) with mild asthma phenotype (35 normal, 18 overweight and 8 obese)	GC-MS	Linoleic, oleic, erucic, cis-11-eicosenoic and arachidic acids significantly associated with poorer asthma control and lung function (FEV1, FVC, FEV1/FVC, PEF, FEF_25–75%_ e FeNO) in overweight/obese children No associations for arachidonic, α-linolenic, EPA and DHA	Fatty acid profiling may support personalized nutrition to enhance asthma control in children
PLASMA [35]	380 asthmatic children	LC-MS	Specific metabolites correlated with three clinical features associated with disease severity: -AHR correlated with 91 of the 574 metabolites -%FEV1/FVC ratio pre- and post-bronchodilator with 102 and 155, respectively Key metabolites: thiamine, creatinine, fatty acids (oleic, myristic), carnitine and gammalinolenic acid	Asthma severity metabolome reflects systemic biological alterations across severity levels, highlighting the potential of metabolomics to refine phenotyping and enable personalized assessment
PLASMA [36]	1.165 asthmatic subjects (aged 6 and 14 years) from 2 different cohorts	LC-MS + SNF and spectral clustering	Detection of 5 metabo-endotypes with significant phenotypic differences, including pre and post bronchodilator FEV1/FVC Key metabolites: cholesterol esters, triglycerides and fatty acids	Reproducible and clinically relevant metabo-endotypes support the use of metabolomics in asthma precision medicine
PLASMA [38]	Asthmatics children 6–17 years (257 lean, 99 overweight, 138 obese)	UPLC-TSQ MS	Specific markers of systemic inflammation in obese children (↑ leptin, CRP and certain amino acid metabolites associated with glutathione/oxidative stress pathway) ↓ concentrations of arginine-related metabolites in uncontrolled obese asthma patients than in obese controlled asthma at 12 months	Persistent symptoms and systemic metabolic inflammation characterize obesity-related asthma, supporting amino acid–based biomarkers for stratified management
SERUM [39]	158 adolescents (39 obese asthmatics, 39 healthy-weight asthmatics, 38 obese controls and 42 healthy-weight controls)	HPLC GC	↓ total carotenoid levels and ↑ n-6/n-3 PUFA ratio in obese asthmatic children, both linked to ↓ FEV1 and ↑ IR	Nutritional modulation may improve asthma outcomes in obese children
SERUM [40]	89 asthmatic children 7–11 years (49, healthy-weight, 40 obese)	Multi-omics integration with SNF (metabolomics WITH NMR, transcriptomics, epigenomics)	Anthropometric, metabolic, nutritional and immune factors contribute interdependently to the obese asthma phenotype WHR and metabolic markers (↑ HOMA-IR, ↑ leptin, ↓ adiponectin) showed the strongest associations with reduced lung function, though none predicted symptom-based severity or control	Truncal adiposity is a key driver of obese asthma, supporting metabolic–immune endotyping
SERUM [41]	602 asthmatic children, 593 controls without asthma.	NMR	↓ levels of citrate, ketone bodies, histidine and glutamine in asthma cases compared to controls Lipid metabolites lost significance after controlling for obesity with the exception of FC% in mVLDL and SFA%	Nutrient gaps linked to asthma mechanisms; potential for dietary-based therapies
SERUM [42]	55 children (27 with asthma and 28 control)	NMR + shotgun metagenomics	Significant microbe–metabolite associations in asthmatic children: ↓ Prevotella sp. oral taxon 306 and ↓ DMG, ↓ dimethylamine, ↓ glucose, ↓ pyridoxine, and ↑ proline-glutamate chimera, ↑ serine, ↑ lactate Several control-enriched species inversely correlated with total and allergen-specific IgE levels	Integration of metagenomics and metabolomics reveals host–microbiome interactions in mite-sensitized pediatric asthma with diagnostic implications
SERUM [21]	53 children, aged 3–5 years, (15 lowly sensitized non-atopic asthma, 13 highly sensitized atopic asthma, 25 healthy controls)	NMR + 16S rRNA sequencing	Tyrosine, isovalerate, glycine and histidine associated with lowly sensitized asthma Acetic acid correlated with highly sensitized asthma and airway microbiota composition	Distinct metabolites associated with IgE profiles and microbiota–asthma axis

Abbreviations: ↑—increased; ↓—decreased; NMR—nuclear magnetic resonance; ML—machine learning; LC-MS—liquid chromatography–mass spectrometry; UHPLC-MS/MS—ultra-high-performance liquid chromatography and tandem mass spectrometry; HPLC-MS—high-performance liquid chromatography–mass spectrometry; MS—mass spectometry; UPLC—ultra-high-performance liquid chromatograph; CRP—C-reactive protein; CS—corticosteroids; DMG—dimethylglycine; FEV1—forced expiratory volume in the first second; ERV—expiratory reserve volume; FVC—forced vital capacity; PEF—peak expiratory flow; PEF_25–75%_—peak expiratory flow of the 25–75% of the pulmonary volume; FeNO—fractional exhaled Nitric Oxid; FRC—functional residual capacity; IC—inspiratory capacity; GC-MS—gas chromatography–mass spectrometry; HPLC—high-performance liquid chromatography; FC% in mVLDL—ratio of free cholesterol to total lipids in the mean very low density lipoprotein; SFA%—percentage of the ratio of saturated fatty acids to total fatty acids; SNF—similarity network fusion; AHR—airway hyperresponsiveness; PUFA—polyunsaturated fatty acids; LC-MS—liquid chromatography–mass spectrometry; HOMA-IR—homeostasis model assessment of insulin resistance; IR—insulin resistance.

**Table 4:** Asthma endotyping metabolomics studies on feces and urine.

Samples	Patients	Technique	Key Findings	Clinical Relevance
FECES [43]	110 asthmatic subjects aged 3–5 years	UHPLC-*MS*/*MS* *+* 16S rRNA sequencing	↑ *Veillonella* and histidine metabolites (carnosine, methyl-histidine, β-alanyl-methyl-histidine) in high wheeze group ↑ sphingolipids (sphinganine, sphingosine, ceramides) in ICS-treated non-responders	Gut microbiome–metabolome signatures linked to wheeze severity and ICS response Fecal histidine and sphingolipid metabolites as potential biomarkers in pediatric asthma
FECES + PLASMA [44]	46 asthmatic children, 4–13 years old (13 normal-weight, 8 overweight, 25 obese)	NMR GC-MS *+* 16S rRNA sequencing	↑ Leptin ↓ plasma acetate in obese allergic asthma phenotype ↑ fecal D-lactate, ↑ D/L lactate ratio, and ↑ plasma creatinine in children with worse asthma outcomes ↓ plasma citrate and ↓ dimethylsulfone in persistent asthma	Metabolic alterations reflect obesity-linked asthma severity Plasma and fecal metabolites support endotype-specific asthma profiling
URINE [45]	30 asthmatic children 6–17 years (15 corticosteroid respondent, 15 CS-nonrespondent)	LC coupled with FTMS	3,6-dihydronicotinic acid, 3-methoxy-4-hydroxyphenyl(ethylene)glycol, 3,4-dihydroxyphenylalanine, γ-glutamylcysteine, cysteinylglycine associated with corticosteroid resistance in pediatric severe asthma Key pathways: tyrosine metabolism, degradation of aromatic compounds and glutathione metabolism	Urine metabolomics identifies non-invasive biomarkers of CS resistance, enabling pathway-specific profiling for personalized treatment in severe pediatric asthma

Abbreviations: ↑—increased; ↓—decreased; NMR—nuclear magnetic resonance; UHPLC-*MS*/*MS*—ultra-high-performance liquid chromatography and tandem mass spectrometry; GC-MS—gas chromatography–mass spectrometry; CS—corticosteroids; ICS—inhaled corticosteroids; LC—liquid chromatography; FTMS—Fourier-transform mass spectrometry.

## Data Availability

No new data were created or analyzed in this study.
